# Hydrogen 4-ammonio­phenyl­phospho­n­ate

**DOI:** 10.1107/S1600536811055218

**Published:** 2012-01-07

**Authors:** Kerstin Thiele, Christoph Wagner, Kurt Merzweiler

**Affiliations:** a Institut für Chemie, Naturwissenschaftliche Fakulät II, Martin-Luther-Universität Halle-Wittenberg, Kurt-Mothes-Strasse 2, 06120 Halle, Germany

## Abstract

The title compound, C_6_H_8_NO_3_P, is isostructural with *p*-arsanilic acid. It exists as the zwitterion H_3_N^+^C_6_H_4_PO_3_H^−^. In the crystal, mol­ecules are linked by O—H⋯O and N—H⋯O hydrogen-bond bridges, giving a three-dimensional network structure. The strongest hydrogen bonds are formed between adjacent PO_3_H groups with O⋯O distances of 2.577 (2) Å.

## Related literature

For the synthesis of 4-amino­phenyl­phospho­nic acid, see: Cooper *et al.* (2006 ▶). For the crystal structure of *p*-arsanilic acid, see: Nuttall & Hunter (1996 ▶). For a description of the *TOPOS* program, see: Blatov & Proserpio (2009 ▶). For graph-set descriptors of hydrogen bonds, see: Bernstein *et al.* (1995 ▶)*.* For tables of bond lengths in organic compounds, see: Allen *et al.* (1987 ▶). 
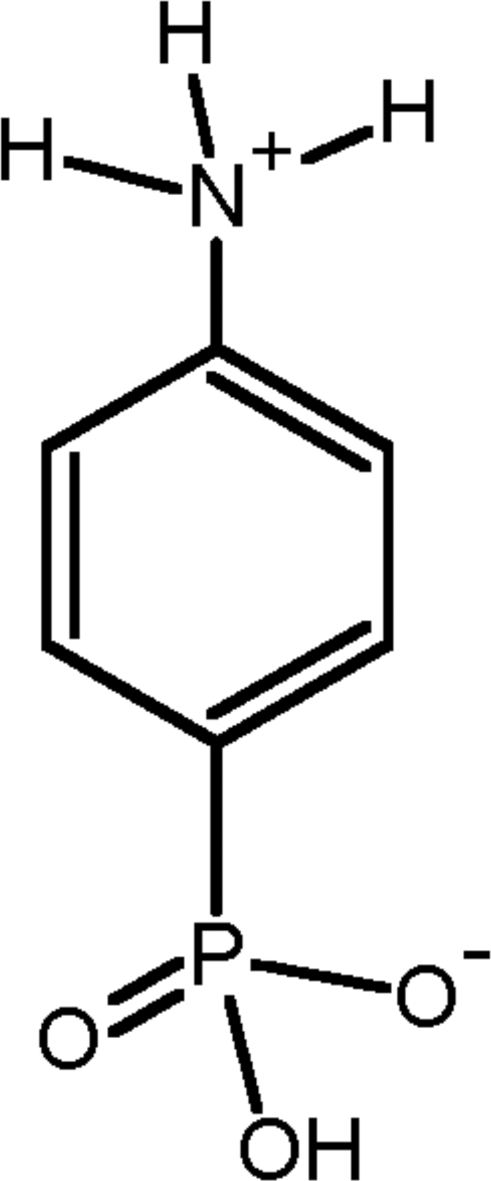


## Experimental

### 

#### Crystal data

C_6_H_8_NO_3_P
*M* *_r_* = 173.10
Monoclinic, 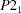

*a* = 7.0967 (13) Å
*b* = 6.2911 (8) Å
*c* = 8.4290 (13) Å
β = 100.606 (14)°
*V* = 369.89 (10) Å^3^
*Z* = 2
Mo *K*α radiation
μ = 0.33 mm^−1^
*T* = 200 K
0.28 × 0.19 × 0.06 mm

#### Data collection

Stoe IPDS 2T diffractometer
2885 measured reflections
1941 independent reflections
1801 reflections with *I* > 2σ(*I*)
*R*_int_ = 0.022

#### Refinement

*R*[*F* ^2^ > 2σ(*F* ^2^)] = 0.029
*wR*(*F* ^2^) = 0.063
*S* = 1.08
1941 reflections
116 parameters
4 restraints
H atoms treated by a mixture of independent and constrained refinement
Δρ_max_ = 0.30 e Å^−3^
Δρ_min_ = −0.24 e Å^−3^
Absolute structure: Flack (1983 ▶), 864 Friedel pairs
Flack parameter: 0.13 (8)

### 

Data collection: *X-AREA* (Stoe & Cie, 2009 ▶); cell refinement: *X-AREA*; data reduction: *X-RED* (Stoe & Cie, 2009 ▶); program(s) used to solve structure: *SHELXS97* (Sheldrick, 2008 ▶); program(s) used to refine structure: *SHELXL97* (Sheldrick, 2008 ▶); molecular graphics: *DIAMOND* (Brandenburg, 2009 ▶); software used to prepare material for publication: *SHELXL97* and *PLATON* (Spek, 2009 ▶).

## Supplementary Material

Crystal structure: contains datablock(s) I, global. DOI: 10.1107/S1600536811055218/vm2144sup1.cif

Structure factors: contains datablock(s) I. DOI: 10.1107/S1600536811055218/vm2144Isup2.hkl

Supplementary material file. DOI: 10.1107/S1600536811055218/vm2144Isup3.cml

Additional supplementary materials:  crystallographic information; 3D view; checkCIF report

## Figures and Tables

**Table 1:** Hydrogen-bond geometry (Å, °)

*D*—H⋯*A*	*D*—H	H⋯*A*	*D*⋯*A*	*D*—H⋯*A*
O3—H4⋯O1^i^	0.95 (3)	1.64 (3)	2.5772 (17)	166 (3)
N—H1⋯O2^ii^	0.92 (2)	1.83 (2)	2.7459 (19)	172 (2)
N—H2⋯O1^iii^	0.93 (2)	1.83 (2)	2.751 (2)	170 (2)
N—H3⋯O2^iv^	0.91 (2)	1.78 (2)	2.692 (2)	178 (3)

Symmetry codes: (i) 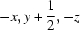; (ii) 
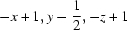
; (iii) 
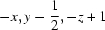
; (iv) 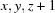.

**Fig. 1.**
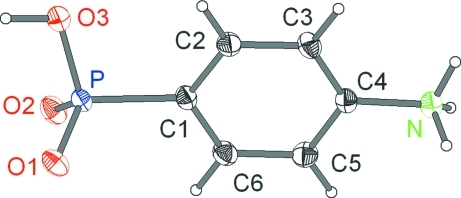
Molecular structure of (I). Thermal ellipsoids are drawn at the 50% probability level.

**Fig. 2.**
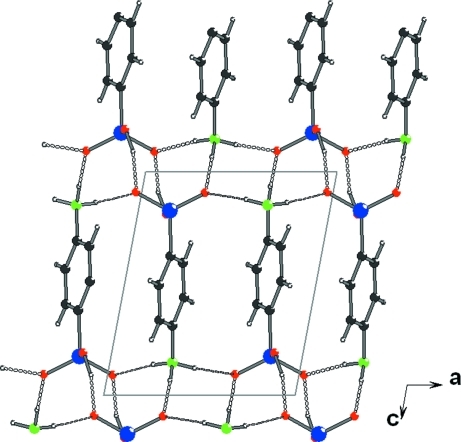
Packing diagram of (I) displaying the hydrogen bond network.

## supplementary crystallographic information

### Comment

Compound (I) is isostructural to the corresponding arsenic derivative *p*-arsanilic acid (Nuttall & Hunter, 1996). Like in the case of the arsenic derivative, compound (I) exists in the form of zwitter ions H_3_N^+^C_6_H_4_PO_3_H^-^, *i.e. p*-ammoniophenylphosphonate. Phosphorus is coordinated nearly tetrahedrally by three O atoms and the carbon atom of the aryl group. The bond lengths between phophorus and the terminal oxygen atoms O1 and O2 are found to be shorter (1.517 (1) and 1.511 (1) Å) than the P—OH bond (1.569 (1) Å). This is in agreement with the orbservation in *p*-arsanilic acid with As—O bonds of 1.656 (6), 1.669 (6) and 1.737 (8) Å. The C—N bond legth of 1.465 (2) Å is essentially the same as in *p*-arsanilic acid (1.479 (10) Å). This is a typical value for C_aryl_ NH_3_^+^ distances (Allen *et al.*, 1987).

The zwitterions are linked by two different types of hydrogen bonds (Table 1). The strongest hydrogen bonds are observed in the case of O—H..O bridges that are formed between adjacing PO_3_H units. Consequently chains with C1,1(4) motifs are formed. Additionally there are N—H···O hydrogen bridges, that are formed between ammonium nitrogen atoms as donors and phosphonate oxygen atoms as acceptors. In this case C1,1(8) structural motifs are found (Bernstein *et al.*, 1995).

As a result of the linkage of NH_3_^+^ and PO_3_H^-^ groups by hydrogen bonds puckered 6^3^ nets are formed. A further (covalent) linkage of the NH_3_^+^ and PO_3_H^-^ groups by C_6_H_4_ units, which act as a kind of pillars between the NH_3_^+^-PO_3_H^-^ layers, leads to a three-dimensional network. This network contains O atoms as 3- c nodes and P and N atoms as 4- c nodes. According to a topological analysis using TOPOS the three-dimensional net can be described by the Schläfli symbol {6^3^.8^2^.10}{6^3^.8^3^}{6^3^}^2^ (Blatov & Proserpio, 2009).

### Experimental

4-aminophenylphosphonic acid was synthesized according to a published procedure by Cooper *et al.* (2006). Single crystals were obtained by recrystallization from hot water.

### Refinement

H atoms bonded to C were placed in calculated positions with a C—H distance of 0.95 Å, *U*_iso_(H)= 1.2*U*_eq_(C). H atoms bonded to N were located from difference fourier maps and refined with N—H distances fixed in the range of 0.91–0.93 Å, *U*_iso_(H) were refined freely. The H atom attached to the phosphonate O atom was located from the difference fourier map and refined freely.

### Crystal data

**Table:**

C_6_H_8_NO_3_P	*Z* = 2
*M**_r_* = 173.10	*F*(000) = 180
Monoclinic, *P*2_1_	*D*_x_ = 1.554 Mg m^−^^3^
Hall symbol: P 2yb	Mo *K*α radiation, λ = 0.71073 Å
*a* = 7.0967 (13) Å	µ = 0.33 mm^−^^1^
*b* = 6.2911 (8) Å	*T* = 200 K
*c* = 8.4290 (13) Å	Plate, colourless
β = 100.606 (14)°	0.28 × 0.19 × 0.06 mm
*V* = 369.89 (10) Å^3^	

### Data collection

**Table:**

Stoe IPDS 2T diffractometer	1801 reflections with *I* > 2σ(*I*)
Radiation source: fine-focus sealed tube	*R*_int_ = 0.022
graphite	θ_max_ = 29.1°, θ_min_ = 2.5°
Detector resolution: 6.67 pixels mm^-1^	*h* = −9→9
rotation method scans	*k* = −8→8
2885 measured reflections	*l* = −11→11
1941 independent reflections	

### Refinement

**Table:**

Refinement on *F*^2^	Secondary atom site location: difference Fourier map
Least-squares matrix: full	Hydrogen site location: inferred from neighbouring sites
*R*[*F*^2^ > 2σ(*F*^2^)] = 0.029	H atoms treated by a mixture of independent and constrained refinement
*wR*(*F*^2^) = 0.063	*w* = 1/[σ^2^(*F*_o_^2^) + (0.0348*P*)^2^ + 0.0226*P*] where *P* = (*F*_o_^2^ + 2*F*_c_^2^)/3
*S* = 1.08	(Δ/σ)_max_ = 0.002
1941 reflections	Δρ_max_ = 0.30 e Å^−^^3^
116 parameters	Δρ_min_ = −0.23 e Å^−^^3^
4 restraints	Absolute structure: Flack (1983), 864 Friedel pairs
Primary atom site location: structure-invariant direct methods	Flack parameter: 0.13 (8)

### Special details

**Table:**

Geometry. All e.s.d.'s (except the e.s.d. in the dihedral angle between two l.s. planes) are estimated using the full covariance matrix. The cell e.s.d.'s are taken into account individually in the estimation of e.s.d.'s in distances, angles and torsion angles; correlations between e.s.d.'s in cell parameters are only used when they are defined by crystal symmetry. An approximate (isotropic) treatment of cell e.s.d.'s is used for estimating e.s.d.'s involving l.s. planes.
Refinement. Refinement of *F*^2^ against ALL reflections. The weighted *R*-factor *wR* and goodness of fit *S* are based on *F*^2^, conventional *R*-factors *R* are based on *F*, with *F* set to zero for negative *F*^2^. The threshold expression of *F*^2^ > σ(*F*^2^) is used only for calculating *R*-factors(gt) *etc*. and is not relevant to the choice of reflections for refinement. *R*-factors based on *F*^2^ are statistically about twice as large as those based on *F*, and *R*- factors based on ALL data will be even larger.

### Fractional atomic coordinates and isotropic or equivalent isotropic displacement parameters (Å^2^)

**Table:**

	*x*	*y*	*z*	*U*_iso_*/*U*_eq_	
P	0.16365 (5)	0.66144 (6)	0.17272 (4)	0.01583 (9)	
O2	0.33057 (17)	0.6022 (2)	0.09314 (14)	0.0225 (3)	
O1	−0.02778 (17)	0.5711 (2)	0.09090 (14)	0.0214 (3)	
O3	0.1487 (2)	0.9089 (2)	0.19018 (14)	0.0243 (3)	
H4	0.086 (5)	0.972 (5)	0.091 (4)	0.065 (10)*	
N	0.3279 (2)	0.3192 (2)	0.85315 (16)	0.0188 (3)	
H1	0.446 (3)	0.256 (4)	0.878 (3)	0.030 (6)*	
H2	0.229 (3)	0.226 (3)	0.862 (3)	0.032 (7)*	
H3	0.332 (4)	0.417 (4)	0.933 (3)	0.040 (7)*	
C4	0.2901 (2)	0.4096 (3)	0.69025 (18)	0.0167 (3)	
C6	0.2847 (2)	0.3629 (3)	0.4075 (2)	0.0196 (3)	
H6A	0.3068	0.2772	0.3199	0.024*	
C1	0.2134 (2)	0.5699 (3)	0.37885 (18)	0.0169 (3)	
C5	0.3230 (2)	0.2830 (3)	0.56352 (19)	0.0199 (3)	
H5A	0.3713	0.1428	0.5833	0.024*	
C3	0.2190 (2)	0.6139 (3)	0.66533 (18)	0.0197 (4)	
H3A	0.1967	0.6983	0.7535	0.024*	
C2	0.1805 (2)	0.6939 (3)	0.50798 (18)	0.0188 (4)	
H2A	0.1316	0.8340	0.4889	0.023*	

### Atomic displacement parameters (Å^2^)

**Table:**

	*U*^11^	*U*^22^	*U*^33^	*U*^12^	*U*^13^	*U*^23^
P	0.01598 (16)	0.01927 (19)	0.01195 (14)	−0.00100 (19)	0.00178 (11)	−0.00142 (18)
O2	0.0202 (5)	0.0307 (8)	0.0176 (5)	−0.0028 (5)	0.0059 (4)	−0.0041 (4)
O1	0.0173 (6)	0.0266 (7)	0.0190 (5)	−0.0008 (5)	−0.0003 (4)	−0.0048 (5)
O3	0.0343 (7)	0.0199 (7)	0.0169 (6)	−0.0007 (6)	−0.0002 (5)	0.0001 (5)
N	0.0178 (7)	0.0227 (9)	0.0157 (6)	0.0003 (5)	0.0023 (5)	0.0020 (5)
C4	0.0135 (6)	0.0212 (8)	0.0150 (6)	−0.0018 (6)	0.0019 (5)	0.0016 (6)
C6	0.0225 (8)	0.0195 (8)	0.0169 (7)	0.0016 (6)	0.0039 (6)	−0.0029 (6)
C1	0.0150 (7)	0.0214 (8)	0.0138 (7)	−0.0021 (6)	0.0018 (5)	0.0003 (6)
C5	0.0206 (7)	0.0184 (8)	0.0210 (7)	0.0024 (7)	0.0045 (6)	−0.0002 (7)
C3	0.0218 (7)	0.0224 (11)	0.0155 (6)	0.0014 (6)	0.0049 (6)	−0.0022 (6)
C2	0.0209 (7)	0.0174 (10)	0.0180 (7)	0.0018 (6)	0.0033 (5)	−0.0007 (6)

### Geometric parameters (Å, °)

**Table:**

P—O2	1.5114 (13)	C4—C5	1.386 (2)
P—O1	1.5165 (13)	C6—C5	1.387 (2)
P—O3	1.5692 (14)	C6—C1	1.402 (3)
P—C1	1.8026 (16)	C6—H6A	0.9500
O3—H4	0.95 (3)	C1—C2	1.393 (2)
N—C4	1.465 (2)	C5—H5A	0.9500
N—H1	0.918 (17)	C3—C2	1.398 (2)
N—H2	0.928 (17)	C3—H3A	0.9500
N—H3	0.908 (18)	C2—H2A	0.9500
C4—C3	1.383 (2)		
			
O2—P—O1	114.54 (7)	C5—C6—C1	120.08 (16)
O2—P—O3	110.99 (8)	C5—C6—H6A	120.0
O1—P—O3	110.14 (8)	C1—C6—H6A	120.0
O2—P—C1	108.58 (8)	C2—C1—C6	119.51 (15)
O1—P—C1	108.57 (8)	C2—C1—P	122.87 (14)
O3—P—C1	103.37 (8)	C6—C1—P	117.60 (12)
P—O3—H4	111 (2)	C4—C5—C6	119.41 (17)
C4—N—H1	112.4 (15)	C4—C5—H5A	120.3
C4—N—H2	108.1 (15)	C6—C5—H5A	120.3
H1—N—H2	112 (2)	C4—C3—C2	118.74 (15)
C4—N—H3	114.3 (18)	C4—C3—H3A	120.6
H1—N—H3	103 (2)	C2—C3—H3A	120.6
H2—N—H3	107 (2)	C1—C2—C3	120.55 (16)
C3—C4—C5	121.71 (15)	C1—C2—H2A	119.7
C3—C4—N	120.13 (15)	C3—C2—H2A	119.7
C5—C4—N	118.15 (16)		
			
C5—C6—C1—C2	−0.3 (3)	C3—C4—C5—C6	0.3 (2)
C5—C6—C1—P	−179.11 (13)	N—C4—C5—C6	178.67 (16)
O2—P—C1—C2	135.65 (14)	C1—C6—C5—C4	0.0 (3)
O1—P—C1—C2	−99.23 (15)	C5—C4—C3—C2	−0.3 (2)
O3—P—C1—C2	17.73 (16)	N—C4—C3—C2	−178.62 (14)
O2—P—C1—C6	−45.59 (15)	C6—C1—C2—C3	0.3 (2)
O1—P—C1—C6	79.53 (14)	P—C1—C2—C3	179.06 (13)
O3—P—C1—C6	−163.52 (13)	C4—C3—C2—C1	0.0 (2)

### Hydrogen-bond geometry (Å, °)

**Table:**

*D*—H···*A*	*D*—H	H···*A*	*D*···*A*	*D*—H···*A*
O3—H4···O1^i^	0.95 (3)	1.64 (3)	2.5772 (17)	166 (3)
N—H1···O2^ii^	0.92 (2)	1.83 (2)	2.7459 (19)	172 (2)
N—H2···O1^iii^	0.93 (2)	1.83 (2)	2.751 (2)	170 (2)
N—H3···O2^iv^	0.91 (2)	1.78 (2)	2.692 (2)	178 (3)

Symmetry codes: (i) −*x*, *y*+1/2, −*z*; (ii) −*x*+1, *y*−1/2, −*z*+1; (iii) −*x*, *y*−1/2, −*z*+1; (iv) *x*, *y*, *z*+1.
